# Association between dietary fiber intake and cancer cachexia: mediation by inflammatory biomarkers

**DOI:** 10.3389/fnut.2026.1757969

**Published:** 2026-02-05

**Authors:** Yan Guo, Caiyan Zhang, Haiyue Wang, Hongmei Xue, Ying Xie, Hongzhen Du, Zengning Li

**Affiliations:** 1 School of Public Health, Hebei Medical University, Shijiazhuang, China; 2 Department of Clinical Nutrition, The First Hospital of Hebei Medical University, Shijiazhuang, China; 3 Hebei Province Key Laboratory of Nutrition and Health, Shijiazhuang, China

**Keywords:** cancer cachexia, dietary fiber intake, inflammation, mediation analysis, nutritional metabolic diseases

## Abstract

**Background:**

Cancer cachexia (CC) is a major cause of death in cancer patients, with chronic inflammation being a key driver. Dietary fiber, a nutrient with strong anti-inflammatory potential, is closely linked to mortality risk in cancer patients. However, the association between dietary fiber intake and cachexia risk remains unclear.

**Method:**

This study collected dietary and clinical data from cancer patients enrolled in the “Investigation for Current States of Dietary Intake and Its Influencing Factors in Patients with Common Cancers” (DIIFC). First, we analyzed the association between dietary fiber intake and cancer cachexia using restricted cubic splines (RCSs). Next, we used multivariable logistic regression models to analyze the relationships between dietary fiber intake, inflammatory markers, and cancer cachexia. Finally, we performed mediation analysis to explore whether inflammation mediates the effect of dietary fiber intake on cancer cachexia.

**Results:**

Of the 720 participants, 198 were diagnosed with cancer cachexia. RCSs revealed a nonlinear inverse correlation between dietary fiber intake and the risk of cancer cachexia (*p* < 0.001). Logistic regression analysis showed that greater dietary fiber intake was associated with a lower risk of cachexia (OR = 0.92, 95% CI: 0.87–0.98, *p* = 0.007). Higher levels of inflammatory markers (WBC, NEU, and NLR) were associated with a greater risk of cachexia (*p* < 0.05). Mediation analysis indicated that WBC, NEU, and NLR significantly mediated the relationship between dietary fiber and cachexia, accounting for 5.67%, 7.62%, and 7.78%, respectively (*p* < 0.05).

**Conclusion:**

Increased dietary fiber intake may exert a protective effect against cancer cachexia, partially mediated through inflammatory pathways. Further exploration of specific fiber subtypes and additional mechanisms is warranted.

## 1 Introduction

Malignant neoplasms are a major contributor to the global disease burden, with cancer cachexia implicated in nearly 40% of cancer-related deaths and prevalent in 33% of patients (1, 2). The incidence of CC varies by tumor type, reaching 60–80% in gastroesophageal and pancreatic cancers, but only 20% in breast and prostate cancers (3, 4). Chronic inflammation, driven by cytokines like TNF-*α* and IL-6, underlies its pathogenesis, causing muscle and fat loss and anorexia (5). Despite some treatments, no effective interventions or approved drugs can reverse cancer cachexia, leaving nutritional support as the mainstay for symptom relief (6–8). Nutritional support for cancer cachexia patients primarily involves energy- and protein-dense formulations, with some benefit from specific nutrients like *ω*-3 fatty acids, BCAAs, and HMB, as well as phytocompounds with antioxidant and anti-inflammatory effects (9–11). However, conventional therapy remains limited, highlighting the need for precision nutrition strategies.

Dietary fiber, an indigestible plant component, reduces disease risk by modulating the microbiota-host interaction and is linked to lower mortality in cancer survivors, with each unit increase in intake reducing cancer-specific mortality by 3.5% (12). It also lowers inflammatory biomarkers like WBC, NEU, and NLR, which are associated with cancer cachexia progression (13, 14). Nevertheless, few population-based studies have investigated the relationship between dietary fiber intake and cancer cachexia.

This study explores the association between dietary fiber intake and cancer cachexia, investigating whether inflammatory biomarkers mediate this relationship, to inform dietary guidance and advance precision nutrition therapy for affected populations.

## 2 Materials and methods

### 2.1 Study design and population

Participants were enrolled in the multicenter, cross-sectional Dietary Intake and Influencing Factors in Common Cancers (DIIFC) study (ChiCTR1900022514), which ran from March to December 2019. Patients with histologically confirmed malignancies, including but not limited to breast, lung, colorectal, gastroesophageal, and liver cancers, as well as gynecological malignancies and lymphoma, were eligible for inclusion. We excluded those with missing data on age, height, weight, dietary survey, tumor stage, or inflammatory biomarkers, and those with active infection. The exclusion criterion of “active infection” was applied based on a comprehensive clinical assessment. Patients were considered to have “active infection” and were excluded if they met any one of the following conditions: (a) presence of definitive infection-related clinical signs, or (b) imaging findings indicative of an active infectious lesion, or (c) confirmed presence of a pathogenic microorganism. The ethics committee of the First Hospital of Hebei Medical University approved the study (approval No. 2013205). Conducted in accordance with the Declaration of Helsinki, the study required written informed consent from all participants.

### 2.2 Assessment of dietary intake

Dietary intake was assessed using a 24-h dietary recall administered by trained research dietitians. To facilitate accurate portion size estimation, participants were assisted with a standardized set of food models, household measuring utensils, and a food photograph atlas during the interview. Energy, nutrients, and other components were calculated with the 6th edition of the *China Food Composition*, using the types and amounts of all foods and beverages consumed in the preceding 24 h. In this study, we extracted data on energy (KJ [kcal]), protein (g), and dietary fiber (g). Three-day means served as the estimate of daily intake.

### 2.3 Measurement of inflammatory biomarkers

Peripheral blood cell count data, including leukocyte, neutrophil, and lymphocyte data, were collected. In accordance with previous studies, the inflammatory biomarkers of the participants in the examination were white blood cell count, neutrophil count, and neutrophil-to-lymphocyte ratio (15, 16). The unit of lymphocyte, neutrophil, and platelet counts was 10^9 cells/L.

### 2.4 Definition for cancer cachexia

According to the 2011 Cachexia Consensus, cachexia is defined on the basis of the following criteria: (1) weight loss greater than 5%, (2) weight loss greater than 2% in individuals already showing depletion according to current bodyweight and height (BMI < 20 kg/m^2^) or skeletal muscle mass (sarcopenia). The criteria for muscle depletion were as follows: appendicular skeletal muscle mass index (ASMI) < 7.26 kg/m^2^ for men and <5.45 kg/m^2^ for women (17). In our study, appendicular skeletal muscle mass (ASM) was estimated by an anthropometric equation, which has been validated in Chinese individuals and demonstrates good agreement with ASM measured by dual-energy X-ray absorptiometry (DXA) (18). The validation study reported a high agreement between the predicted ASM from this equation and the ASM measured by DXA (*r* = 0.941).


$$\left[\begin{array}{l} \mathrm{ASM}=0.193\times \text{body weight}+0.107\times \text{height}\\ -4.157\times \mathrm{sex}-0.037\times \mathrm{age}-2.631 \end{array}\right]$$

In this model, ASM is in kilograms, height is in centimeters, age is in years and sex is represented by 1 (male) or 2 (female). The ASMI was further calculated by dividing the ASM by the height squared in meters (ASM/height^2^).

### 2.5 Covariates

To control confounding, we adjusted for age, sex, BMI, tumor type, stage, and treatment. BMI was categorized as underweight (<18.5), normal (18.5 ~ 23.9), or overweight/obese (≥24.0). Because anorexia—a hallmark of cancer cachexia—reduces total energy intake (19), we also adjusted for energy. Additionally, Protein intake was added because higher intake can counteract catabolism and attenuate muscle loss (20). Dietary protein intake was expressed in g/day.

### 2.6 Statistical analysis

SPSS 26.0 and R software were employed for the statistical analyses. Baseline characteristics were compared after checking normality. In this study, the normality of continuous variables was assessed using the Kolmogorov–Smirnov test, which is appropriate for our sample size (*n* = 720). Normal data are shown as mean ± SD, non-normal as median [IQR], and categorical variables as *n* (%); comparisons used *t*-test, Mann–Whitney U, or χ^2^, respectively. See above; continuous variables were compared with *t*-test or Mann–Whitney U, as appropriate.

The non-linear association between dietary fiber intake and cachexia risk were explored using restricted cubic splines with 4 knots placed at default percentiles. Multivariate logistic regression analyses were performed to examine associations of dietary fiber intake and inflammatory biomarkers with cancer cachexia, with results expressed as odds ratios (ORs) and 95% confidence intervals (CIs). Multivariable logistic regression models were constructed with hierarchical adjustment as follows: Model 1: unadjusted; Model 2: adjusted for age, sex, BMI; Model 3: further adjusted for dietary energy intake and dietary protein intake based on Model 2; Model 4: further adjusted for tumor type, stage and therapy based on Model 3. All covariates within a given model were included simultaneously. The relationships between dietary fiber intake and inflammatory biomarkers were investigated via Spearman correlation analysis. Sensitivity analyses excluded the highest and lowest 5% of fiber intake. Mediation analysis was conducted via R mediation packages to investigate the potential mediating effects of inflammatory biomarkers. The significance of indirect mediation effects was tested using the bootstrap method with 5,000 resamples to derive bias-corrected 95% confidence intervals. *p* < 0.05 indicated a significant difference.

## 3 Results

### 3.1 Characteristics of participants

**Figure 1:**
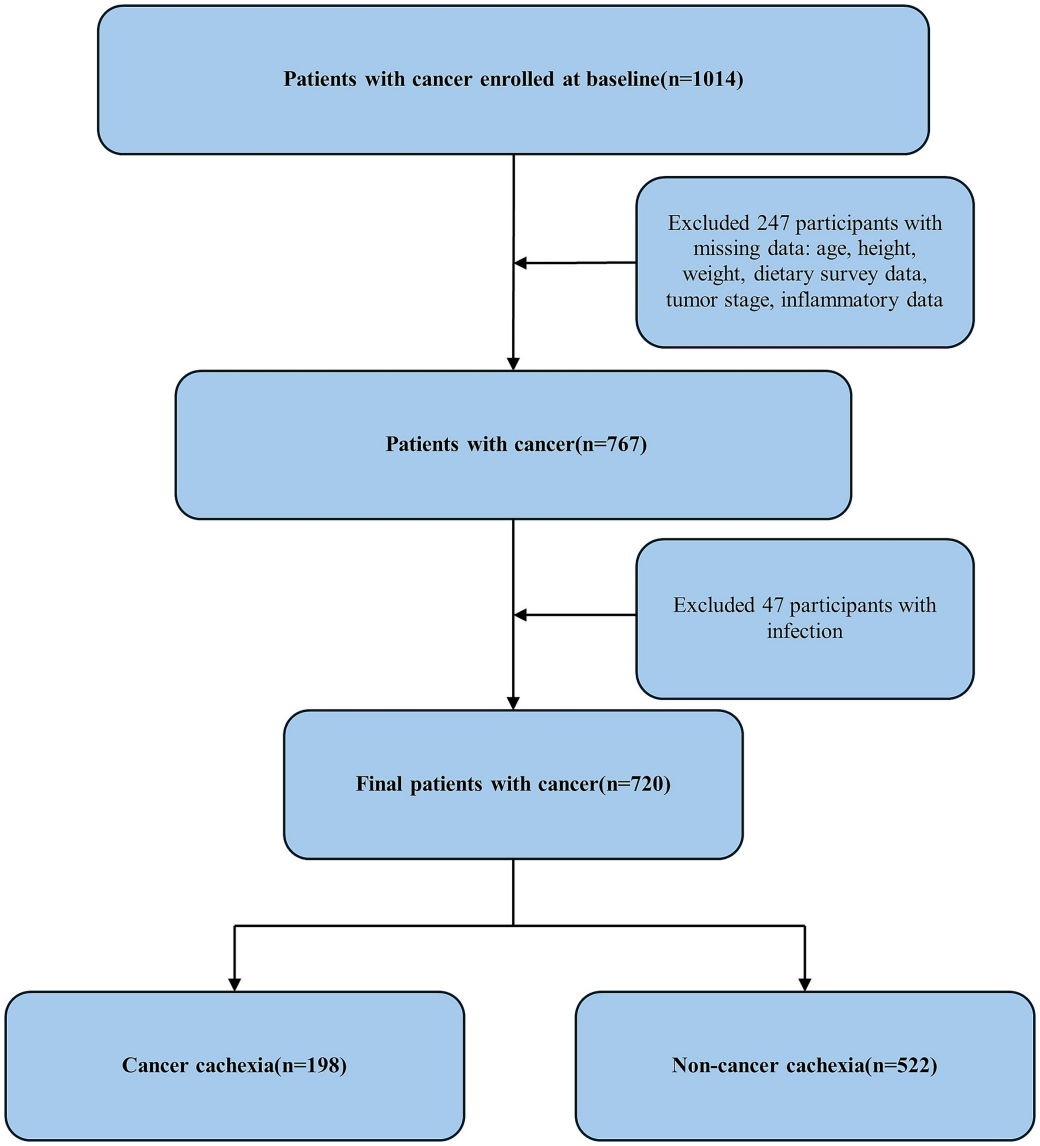
Flowchart of the study design and participants excluded from the study.

A total of 720 patients with cancer were eligible for our final analysis by using the above exclusion criteria (Figure 1). Among them, 198 participants were diagnosed with cancer cachexia. The mean age of the total participants was 65.45 ± 11.78 years, and 60% of the participants were male. Table 1 shows the characteristics of the baseline samples classified by cachexia status. The patients with cancer cachexia were older, and there was a greater proportion of male patients than female patients. Malignant tumor patients consistently demonstrate inadequate dietary fiber intake compared with the recommendations, with a median intake of 6.24 g. More specifically, patients with cancer cachexia had significantly lower dietary fiber, total energy and protein intakes compared to those in the non-cancer cachexia group. Moreover, cachectic individuals presented a greater prevalence of lung cancer, gastroesophageal cancer, and advanced tumor stages, as did a greater proportion of patients who had undergone chemotherapy. However, fewer individuals in the cachexia group were classified as overweight or obese. Additionally, significantly elevated levels of inflammatory biomarkers were observed in the cachexia cohort relative to the non-cachexia controls.

**Table 1:** Clinical characteristics of participants according to cancer cachexia status (*N* = 720).

Characteristics	Total	cancer cachexia	Non-cancer cachexia	*p* value
	*N* = 720	*N* = 198	*N* = 522	
Age	66.90 [58.69, 73.08]	67.74 [59.63, 75.12]	66.61 [58.38, 72.33]	0.022*
Sex				0.016*
Male (%)	432 (60.00)	133 (67.17)	299 (57.28)	
Female (%)	288 (40.00)	65 (32.83)	223 (42.72)	
Tumor type				0.001**
Breast cancer (%)	76 (10.60)	6 (3.03)	70 (13.41)	
Lung cancer (%)	150 (20.80)	37 (18.69)	113 (21.65)	
Liver cancer (%)	30 (4.20)	11 (5.56)	19 (3.64)	
Gastroesophageal cancer (%)	172 (23.90)	62 (31.31)	110 (21.07)	
Lymphoma (%)	41 (5.70)	13 (6.57)	28 (5.36)	
Gynecological cancer (%)	37 (5.10)	11 (5.56)	26 (4.98)	
Colorectal cancer (%)	117 (16.30)	27 (13.64)	90 (17.24)	
Other	97 (13.50)	31 (15.66)	66 (12.64)	
Therapy				0.016*
Surgery (%)	109 (15.14)	32 (16.16)	77 (14.75)	
Chemotherapy (%)	431 (59.86)	113 (57.07)	318 (60.92)	
Radiotherapy (%)	65 (9.03)	28 (14.14)	37 (7.09)	
Other (%)	115 (15.97)	25 (12.63)	90 (17.24)	
Tumor stage				0.089
I (%)	27 (3.75)	5 (2.53)	22 (4.21)	
II (%)	170 (23.61)	38 (19.19)	132 (25.29)	
III (%)	186 (25.83)	62 (31.31)	124 (23.75)	
IV (%)	337 (46.81)	93 (46.97)	244 (46.74)	
BMI (kg/m^2^)				<0.001***
<18.5 (%)	105 (14.60)	58 (29.29)	47 (9.00)	
18.5 ~ 23.9 (%)	375 (52.10)	109 (55.05)	266 (50.96)	
≥24.0 (%)	240 (33.30)	31 (15.66)	209 (40.04)	
WBC (10^9cells/L)	5.63 [4.33 ~ 7.30]	6.22 [4.60 ~ 8.50]	5.52 [4.28 ~ 7.00]	0.001**
NEU (10^9cells/L)	3.63 [2.55 ~ 5.22]	4.34 [2.98 ~ 6.25]	3.46 [2.46 ~ 4.85]	<0.001***
LYM (10^9cells/L)	1.22 [0.83 ~ 1.69]	1.01 [0.70 ~ 1.49]	1.29 [0.92 ~ 1.76]	<0.001***
NLR	2.94 [1.81 ~ 5.35]	4.14 [2.28 ~ 8.29]	2.67 [1.70 ~ 4.40]	<0.001***
Dietary fiber intake (g)	6.24 [3.73 ~ 9.23]	4.25 [2.25 ~ 7.24]	6.87 [4.59 ~ 9.73]	<0.001***
Dietary energy intake (KJ [kcal])	4905.14 [1172.36] [3351.84 [801.11] ~ 6520.10 [1558.34]]	4078.44 [974.77] [2457.96 [587.47] ~ 5807.13 [1387.94]]	5100.35 [1219.01] [3806.63 [909.80] ~ 6595.49 [1576.36]]	<0.001***
Dietary protein intake (g)	45.81 [30.67 ~ 58.92]	37.11 [22.98 ~ 53.68]	47.72 [33.48 ~ 60.87]	<0.001***

Values are reported as median [25th, 75th percentiles]. BMI, body mass index; WBC, white blood cell; NEU, neutrophil; LYM, lymphocyte; NLR, neutrophil-to-lymphocyte ratio.

*p value < 0.05, **p value < 0.01, ***p value < 0.001.

### 3.2 Association between dietary fiber intake and cancer cachexia

To explore the relationship between dietary fiber intake and cachexia risk in cancer patients, we employed RCS analysis. The results revealed a significant nonlinear association (*p* < 0.001), which remained statistically significant after full adjustment for covariates (Supplementary Figure S1). Subsequently, multivariate logistic regression analysis, adjusted for all covariates, revealed a significant inverse association between dietary fiber intake and cachexia risk (OR: 0.92; 95% CI: 0.87 ~ 0.98; *p* = 0.007) (Table 2). Based on quartiles of dietary fiber intake (categorized as Q1–Q4, with Q1 as the reference), we observed a decreasing trend in cachexia risk with increasing fiber intake. Patients in the highest intake quartile (Q4) exhibited a 66% lower risk of cachexia compared to those in the lowest quartile (Q1). On the basis of the results of logistic regression analysis and restricted cubic splines, a dietary fiber intake of 6.24 g may suggest a critical risk threshold.

**Table 2:** Association between dietary fiber intake and the risk of cancer cachexia in participants.

Variables	Model 1		Model 2		Model 3		Model 4	
	OR (95% CI)	*p* value	OR (95% CI)	*p* value	OR (95% CI)	*p* value	OR (95% CI)	*p* value
Dietary fiber intake (g)	0.86 (0.82 ~ 0.91)	<0.001***	0.89 (0.85 ~ 0.94)	<0.001***	0.92 (0.86 ~ 0.97)	0.003**	0.92 (0.87 ~ 0.98)	0.007**
Quartile								
Q1 (<3.74)	1.00 (Reference)		1.00 (Reference)		1.00 (Reference)		1.00 (Reference)	
Q2 (3.74 ~ 6.23)	0.44 (0.29 ~ 0.69)	<0.001***	0.45 (0.29 ~ 0.72)	0.001**	0.48 (0.29 ~ 0.78)	0.003**	0.53 (0.32 ~ 0.88)	0.014*
Q3 (6.24 ~ 9.23)	0.23 (0.14 ~ 0.37)	<0.001***	0.29 (0.17 ~ 0.48)	<0.001***	0.32 (0.18 ~ 0.56)	<0.001***	0.36 (0.20 ~ 0.65)	0.001**
Q4 (≥9.24)	0.21 (0.13 ~ 0.34)	<0.001***	0.28 (0.17 ~ 0.47)	<0.001***	0.32 (0.17 ~ 0.60)	<0.001***	0.34 (0.18 ~ 0.65)	0.001**

OR, Odds Ratio; CI, Confidence Interval.

Model 1: unadjusted; Model 2: adjusted for age, sex, BMI; Model 3: further adjusted for dietary energy intake and dietary protein intake based on Model 2; Model 4: further adjusted for tumor type, stage and therapy based on Model 3.

*p value < 0.05, **p value < 0.01, ***p value < 0.001.

### 3.3 Subgroup analysis of the association between dietary fiber intake and cancer cachexia

In further analysis, we stratified the population by age, sex, BMI, type of cancer, tumor stage, and treatment method since these factors may be correlated with risk of cancer cachexia. In this study, the sample sizes of the cachexia group of breast cancer patients and stage I patients were limited. To ensure the robustness of the results, the tumor types were categorized as digestive malignancies or non-digestive malignancies. This classification was based on existing evidence indicating a greater susceptibility to cachexia in patients with digestive malignancies. Furthermore, given the lower incidence rates of cachexia in TNM stage I and II patients, stages I and II were defined as early-stage disease, whereas stage III and above were defined as advanced-stage disease. According to the subgroup analysis, the benefits of dietary fiber intake may be more pronounced in patients aged ≥60 years, those with a BMI of 18.5 ~ 23.9 kg/m^2^, those with non-digestive system cancer, and those with advanced-stage disease (Table 3). More importantly, our subgroup analysis based on patient sex revealed a suggestive difference in the association between dietary fiber intake and the risk of cancer cachexia. The inverse association remained statistically significant in female patients (*p* = 0.006), whereas it was attenuated and not statistically significant in male patients (*p* = 0.188). The *p*-value for interaction was 0.088. While this interaction term did not reach the conventional threshold of statistical significance, the magnitude of difference in effect estimates between sexes and the borderline *p*-interaction value suggest a potential effect modification by sex. This pattern indicates that the protective role of dietary fiber might be more pronounced or primarily observable in female patients within our cohort. Furthermore, a significant interaction effect was observed between tumor type and therapy with dietary fiber intake (*p* = 0.039, and *p* = 0.007, respectively).

**Table 3:** Subgroup analysis of the association between dietary fiber intake and the risk of cancer cachexia.

Variables	*N* (%)	OR (95% CI)	*p* value	*p* for interaction
Age				0.057
<60y	209 (29.03)	1.01 (0.91 ~ 1.12)	0.838	
≥60y	511 (70.97)	0.88 (0.81 ~ 0.95)	0.001**	
Sex				0.088
Male	432 (60.00)	0.95 (0.89 ~ 1.02)	0.188	
Female	288 (40.00)	0.86 (0.77 ~ 0.96)	0.006**	
BMI (kg/m^2^)				0.191
<18.5	105 (14.58)	0.91 (0.74 ~ 1.12)	0.363	
18.5 ~ 23.9	375 (52.08)	0.87 (0.80 ~ 0.94)	0.001**	
≥24.0	240 (33.33)	1.03 (0.93 ~ 1.13)	0.564	
Tumor type				0.039*
Digestive system cancer	319 (44.31)	0.97 (0.90 ~ 1.05)	0.505	
Non-digestive system cancer	401 (55.69)	0.87 (0.79 ~ 0.96)	0.005**	
Stage				0.289
Early stage	27 (3.75)	0.99 (0.89 ~ 1.11)	0.921	
Advanced stage	523 (23.61)	0.92 (0.86 ~ 0.98)	0.012*	
Therapy				0.007*
Surgery	109 (15.14)	0.93 (0.80 ~ 1.08)	0.523	
Chemotherapy	431 (59.86)	0.95 (0.89 ~ 1.02)	0.178	
Radiotherapy	65 (9.03)	0.94 (0.67 ~ 1.32)	0.709	
Other	115 (15.97)	0.87 (0.69 ~ 1.09)	0.246	

OR, Odds Ratio; CI, Confidence Interval.

Tumor types were categorized as digestive system cancer and non-digestive system cancer. Stages I and II were defined as early-stage, while stage III and above were defined as advanced-stage.

*p value < 0.05, **p value < 0.01, ***p value < 0.001.

### 3.4 Sensitivity analysis of the association between dietary fiber intake and cancer cachexia

To validate the robustness of the model, we conducted a sensitivity analysis by excluding the upper and lower 5% of dietary fiber intake. The results are presented in Supplementary Table S1. Multivariate logistic regression analysis showed that dietary fiber intake remained negatively correlated with cachexia risk (OR: 0.88; 95% CI: 0.81 ~ 0.95; *p* = 0.001).

### 3.5 Analysis of the mediating effects of inflammatory markers

In regard to the influence of inflammation, the relationships between the blood inflammatory and immune biomarkers and cachexia were explored. Table 4 demonstrates the association between different inflammatory markers and cancer cachexia. In the fully adjusted logistic regression model, statistical significance was found for three biomarkers: WBC (OR = 1.054, 95% CI: 1.012 ~ 1.098, *p* = 0.012), NEU (OR = 1.070, 95% CI: 1.023 ~ 1.119, *p* = 0.003), and NLR (OR = 1.043, 95% CI: 1.013 ~ 1.074, *p* = 0.005). In addition, the correlation analysis demonstrated that dietary fiber intake was negatively correlated with the levels of WBC, NEU, and NLR, with correlation coefficients of −0.103 (*p* = 0.006), −0.152 (*p* < 0.001), and −0.219 (*p* < 0.001), respectively.

**Table 4:** Association between inflammatory biomarkers and the risk of cancer cachexia in participants.

Variables	Model1		Model2		Model3	
	OR (95% CI)	*p* value	OR (95% CI)	*p* value	OR (95% CI)	*p* value
WBC	1.061 (1.022 ~ 1.102)	0.002**	1.055 (1.013 ~ 1.098)	0.009**	1.054 (1.012 ~ 1.098)	0.012*
NEU	1.082 (1.037 ~ 1.129)	<0.001***	1.073 (1.026 ~ 1.122)	0.002**	1.070 (1.023 ~ 1.119)	0.003**
LYM	0.667 (0.510 ~ 0.870)	0.003**	0.759 (0.582 ~ 0.989)	0.042*	0.823 (0.637 ~ 1.064)	0.138
NLR	1.069 (1.037 ~ 1.103)	<0.001***	1.050 (1.020 ~ 1.082)	0.001**	1.043 (1.013 ~ 1.074)	0.005**

OR, Odds Ratio; CI, Confidence Interval; WBC, white blood cell; NEU, neutrophil; LYM, lymphocyte; NLR, neutrophil-to-lymphocyte ratio.

Model 1: unadjusted; Model 2: adjusted for age, sex, BMI; Model 3: further adjusted for tumor type, stage and therapy based on Model 2.

*p value < 0.05, **p value < 0.01, ***p value < 0.001.

**Figure 2:**
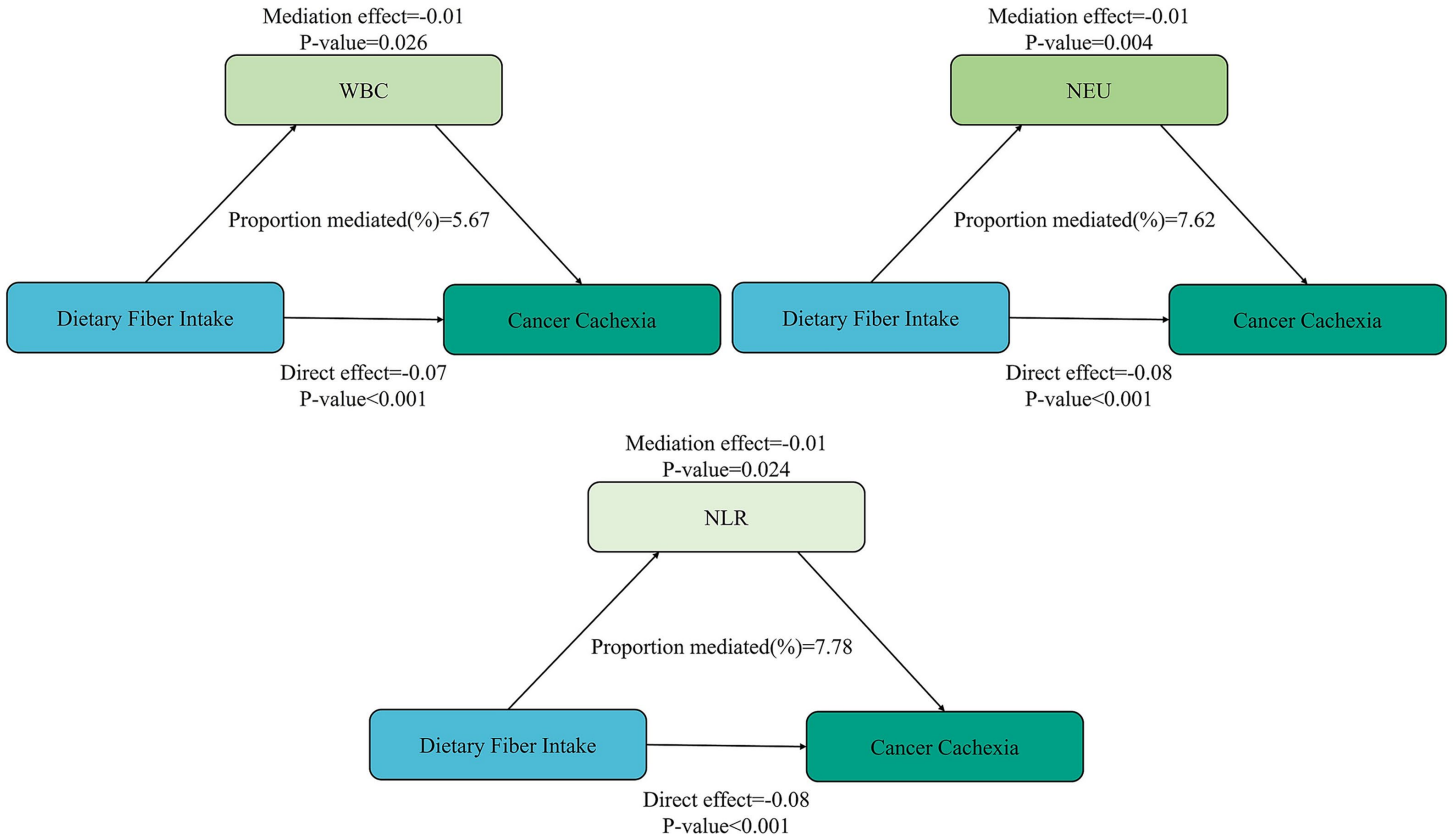
Mediation analysis of inflammatory biomarkers on the relationship between dietary fiber intake and cancer cachexia. WBC, white blood cell; NEU, neutrophil; NLR, neutrophil-to-lymphocyte.

Furthermore, mediation analyses were conducted to explore the mediating effect of inflammatory markers. As shown in Figure 2, all three inflammatory markers significantly mediated the association between dietary fiber intake and cancer cachexia, with WBC, neutrophils, and NLR accounting for 5.67%, 7.62%, and 7.78% of the associations, respectively (*p* < 0.05). Moreover, the direct effects were also significant in these four cases (*p* < 0.001).

## 4 Discussion

Cancer cachexia is a major cause of death in cancer patients and significantly affects their quality of life and treatment outcomes (21). The pathophysiology of cancer cachexia is highly complex, and involves inflammation, metabolic disturbances, energy imbalances, neuroendocrine and appetite dysregulation, and tumor-host interactions (22). These factors are the main reasons why effective treatments are still lacking despite extensive research efforts. Inflammation is widely recognized as the core driver of cachexia (23–25). Recent studies have shown that dietary fiber has the potential to modulate systemic inflammatory responses (26). Therefore, our study focused on the relationship between dietary fiber intake in cancer patients and cancer cachexia. We found that patients with cachexia had significantly lower dietary fiber intake. An increase in dietary fiber intake was associated with a reduced risk of cachexia in a non-linear manner. Further analysis revealed a negative correlation between dietary fiber intake and inflammatory markers, which play a significant mediating role in the relationship between dietary fiber intake and cachexia risk.

Dietary fiber is a macronutrient whose impact on health and disease is increasingly recognized. Our study found that cancer patients have significantly lower dietary fiber intake than the recommended levels in dietary guidelines. In fact, data from the China Health and Nutrition Survey show that the average daily intake of dietary fiber is 19.4 g for men and 17.6 g for women, both of which are lower than the recommended intake of 25–30 g (27, 28). This suggests that the dietary structure of the general population, particularly cancer survivors, may need to be adjusted to increase the intake of grains, tubers, vegetables, and fruits. Shan YJ and Sidahmed E et al. reported that dietary fiber intake is nonlinearly and negatively associated with mortality in cancer patients, which is consistent with our findings that dietary fiber intake is related to cachexia (29, 30). The protective effect of dietary fiber has a “diminishing marginal benefit” characteristic, which is biologically plausible. On the one hand, there are limitations in the transport, metabolism, or storage of dietary fiber. Its beneficial effects may be mediated by fermentable gut bacteria that can become saturated. Extremely high intake of dietary fiber may weaken its benefits by interfering with nutrient absorption or causing gastrointestinal discomfort (31, 32). On the other hand, its protective effect is also constrained by other factors such as population differences, tumor types, and dietary patterns. Interestingly, our study also observed a higher proportion of cachexia in males than in females, and subgroup analysis showed a more significant association between dietary fiber and cancer cachexia in women. This may be due to gender differences in inflammatory responses and hormonal influences (33). For example, estrogen in females can reduce age-related increases in pro-inflammatory cytokines, providing a protective effect on muscle mass (34). What else, differences in gut microbiota composition between males and females are also important contributing factors. The stronger *β*-glucuronidase activity in the female gut microbiota can reactivate conjugated estrogen, promoting M2 macrophages and regulatory B cells, thereby enhancing anti-inflammatory capacity (35). Compared with the non-cachexia group, the cachexia group was older, had more advanced tumors, and consumed less total energy and protein. The aging process may increase susceptibility to cachexia by promoting systemic inflammation and metabolic disturbances (36). More advanced tumor stages signify greater tumor burden, which promotes increased production of pro-inflammatory cytokines, ultimately driving the catabolism of muscle and adipose tissue (37). Reduced energy and protein intake, accelerated protein breakdown, and inhibited protein synthesis promote the development of cachexia (38, 39). Additionally, the incidence of cachexia is greater in patients with gastrointestinal malignancies, especially gastric and esophageal cancers, and there is an interaction with dietary fiber that cancels out its protective effect. This may be due to the patients’ own difficulties in eating and impaired digestive function caused by the disease (40).

More importantly, beyond establishing the association between dietary fiber intake and cachexia, our study provides initial evidence through mediation analysis that dietary fiber may reduce cachexia risk partially through attenuating systemic inflammation. Following fermentation by the gut microbiota (e.g., *Bacteroides genus* and *Bifidobacterium genus*), dietary fiber generates short-chain fatty acids (SCFAs) such as acetate, propionate, and butyrate, which exhibit significant anti-inflammatory effects, improve gut barrier integrity, and modulate muscle metabolism (41–44). Specifically, butyrate inhibits histone deacetylases (HDACs), thereby blocking nuclear translocation of NF-κB and reducing the expression of pro-inflammatory cytokines including TNF-*α* and IL-6 (45–47). Moreover, SCFAs serve as the primary energy substrate for colonic epithelial cells, maintaining intestinal barrier integrity and inhibiting bacterial/endotoxin translocation. This translocation constitutes a key driver of systemic inflammation (48–51). The WBC, NEU, and NLR represent reliable indicators of systemic inflammation and physiological stress. In our study, increased dietary fiber intake was inversely correlated with these inflammatory markers, which aligns with the findings of established studies. Specifically, Jia et al. reported that per unit increase in dietary fiber intake, the WBC decreased by 0.01624 units, the NEU decreased by 0.01346 units, and NLR decreased by 0.00803 units (13). Furthermore, multicenter cohort studies have revealed that cachectic cancer patients exhibit markedly elevated inflammatory biomarker levels, which inversely correlate with overall survival (OS). Notably, NLR > 3.5 serves as an independent predictor of significantly increased mortality risk (HR = 1.22, 95% CI: 1.18 ~ 1.26) after adjusting for key confounders (14). Within the chronic inflammatory milieu, sustained immune cell secretion of pro-inflammatory cytokines—notably TNF-*α* and IL-6—drives muscle catabolism. TNF-α promotes proteolytic degradation through the suppression of insulin signaling pathways and the activation of the ubiquitin-proteasome system (UPS). Concurrently, IL-6 induces muscle atrophy primarily via STAT3 signal transduction (52). Thus, we propose that the protective effect of increased dietary fiber intake against cachexia may operate through a mechanistic pathway involving gut microbiota modulation, intestinal barrier preservation, and subsequent attenuation of systemic inflammation. Our mediation analysis provides statistical support for this hypothesized biological cascade.

Our study has several notable strengths. First, employing restricted cubic splines, we revealed a significant nonlinear inverse association between dietary fiber intake and cachexia risk, suggesting the existence of a critical intake threshold. Most importantly, to our knowledge, this is the first clinical study to statistically demonstrate, through formal mediation analysis, that the protective effect of increased dietary fiber intake against cachexia is significantly mediated by a reduction in systemic inflammation. This finding provides crucial evidence supporting the “dietary fiber-inflammation-cachexia” axis. Furthermore, the observed inverse association remained significant even after rigorous adjustment for key confounders, highlighting the independent role of dietary fiber. Finally, the integration of dietary assessment with readily available clinical inflammatory markers enhances the translational potential of our findings. However, several limitations warrant consideration. The observational nature of our study precludes definitive causal inferences regarding the relationships among dietary fiber, inflammatory biomarkers, and cancer cachexia. Second, potential measurement errors inherent in dietary assessment methods (e.g., recall bias) might have led to misclassification of fiber intake and attenuated the observed associations. Third, we focused on total dietary fiber intake and were unable to differentiate the potential distinct effects of various fiber types or sources (soluble vs. insoluble, cereal vs. fruit/vegetable). Fourth, the lack of longitudinal inflammatory data and other key cytokines limits a more comprehensive understanding of the inflammatory milieu. Finally, while mediation analysis suggests a pathway, it relies on strong assumptions that are difficult to fully satisfy in observational studies, and residual confounding by unmeasured factors (e.g., detailed treatment regimens, physical activity, specific comorbidities) cannot be entirely ruled out. The findings should be interpreted as exploratory, as we did not adjust for multiple comparisons, which increases the risk of Type I error. Future research should conduct randomized controlled trials to investigate whether increasing dietary fiber intake can effectively prevent or delay the occurrence/progression of cachexia. Additionally, studies should examine the specific effects of different types of dietary fiber on cachexia and further explore their underlying mechanisms.

## 5 Conclusion

Our findings suggest that increased dietary fiber intake may exert a protective effect against cancer cachexia. Blood-cell-based inflammatory biomarkers were an easily accessible and cost-effective strategy for identifying cancer cachexia and also significantly mediates the association between dietary fiber intake and cancer cachexia. This study provides preliminary evidence for the association between dietary fiber intake and cancer cachexia, which is mediated through inflammatory pathways. Future well-designed investigations should reveal the effects of specific fiber subtypes, the influence of gut microbiota, and elucidate more potential mechanisms.

## Data Availability

The datasets generated and analyzed during the current study are not publicly available but are available from the corresponding author on reasonable request. Requests to access the datasets should be directed to Zengning Li, zengningli@hebmu.edu.cn.
