# Supplementary material for: Association between dietary fiber intake and cancer cachexia: mediation by inflammatory biomarkers
Source: Front Nutr. 2026 Feb 5;13:1757969. doi: 10.3389/fnut.2026.1757969 (PMC12916402; doi:10.3389/fnut.2026.1757969)
Supplement: Supplementary file 1 [file Table_1.docx]

Supplementary Material

**Table S1.** Sensitivity analysis of the association between dietary fiber intake and the risk of cancer cachexia

| Variables | Model1 | |  | Model2 | |  | Model3 | |  | Model4 | |
| --- | --- | --- | --- | --- | --- | --- | --- | --- | --- | --- | --- |
|  | OR  (95%CI) | *P* value |  | OR  (95%CI) | *P* value |  | OR  (95%CI) | *P* value |  | OR  (95%CI) | *P* value |
| Dietary fiber intake (g) | 0.83  (0.78~0.89) | <0.001*** |  | 0.86  (0.81~0.92) | <0.001*** |  | 0.87  (0.80~0.93) | <0.001*** |  | 0.88  (0.81~0.95) | 0.001** |
| Quartile |  |  |  |  |  |  |  |  |  |  |  |
| Q1(＜3.94) | 1.00 (Reference) |  |  | 1.00 (Reference) |  |  | 1.00 (Reference) |  |  | 1.00 (Reference) |  |
| Q2(3.94~6.23) | 0.43  (0.27 ~ 0.70) | 0.001** |  | 0.45  (0.27 ~ 0.74) | 0.002** |  | 0.45  (0.27~ 0.76) | 0.003** |  | 0.51  (0.30 ~ 0.87) | 0.014* |
| Q3(6.24~8.87) | 0.26  (0.15 ~ 0.43) | <0.001*** |  | 0.33  (0.19 ~ 0.56) | <0.001*** |  | 0.34  (0.19 ~ 0.61) | <0.001*** |  | 0.39  (0.21 ~ 0.72) | 0.002** |
| Q4(≥8.88) | 0.26  (0.13 ~ 0.43) | <0.001*** |  | 0.33  (0.19 ~ 0.58) | <0.001*** |  | 0.35  (0.19 ~ 0.65) | 0.001** |  | 0.38  (0.20 ~ 0.73) | 0.003** |
| OR: Odds Ratio, CI: Confidence Interval  Model 1: unadjusted; Model 2: adjusted for age, sex, BMI; Model 3: further adjusted for dietary energy intake and dietary protein intake based on Model 2; Model 4: further adjusted for tumor type, stage and therapy based on Model 3  * *P* value < 0.05, ** *P* value < 0.01, *** *P* value < 0.001 | | | | | | | | | | | |


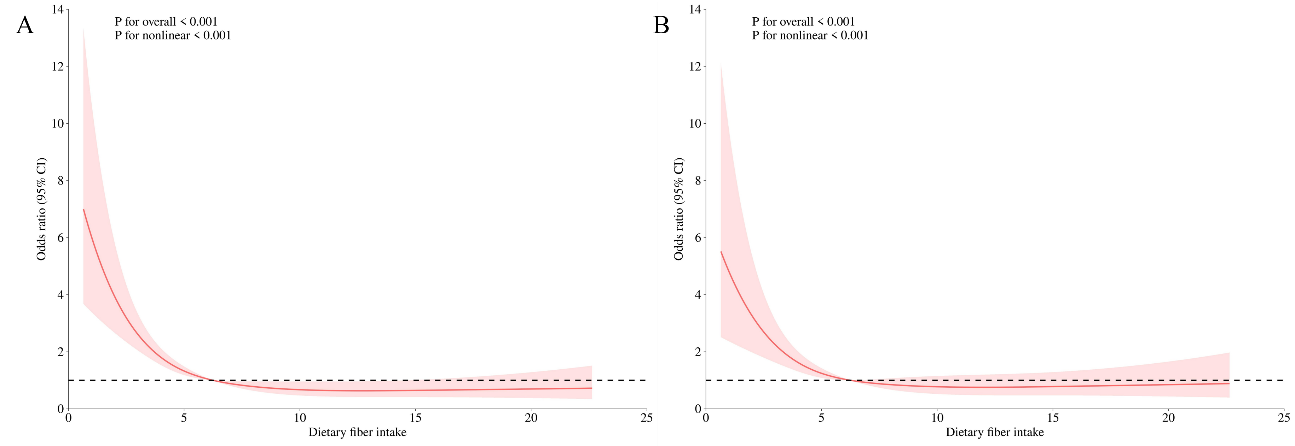


**Figure S1** Association between dietary fiber intake and cancer cachexia. A: Association between dietary fiber intake and cancer cachexia without adjustment. B: Association between dietary fiber intake and cancer cachexia after full adjustment.
